# De Garengeot hernia with intestinal obstruction and appendiceal abscess in an elderly Peruvian patient: a case report

**DOI:** 10.3389/fsurg.2026.1905121

**Published:** 2026-08-12

**Authors:** Alvaro Veleto, Cesar Sapaico-Del-Castillo, Roberto Orta-Barriga, Mijail Vega-Barriga, Carla Cuya-Zevallos

**Affiliations:** 1 Faculty of Human Medicine, Universidad Católica de Santa María, Arequipa, Peru; 2 Faculty of Human Medicine, Universidad Católica de Santa María, Arequipa, Peru; 3 Faculty of Nursing, Universidad Católica de Santa María, Arequipa, Peru

**Keywords:** acute appendicitis, case report, De Garengeot hernia, femoral hernia, intestinal obstruction, older adults

## Abstract

**Background:**

De Garengeot hernia is a rare entity defined by the presence of the vermiform appendix within the sac of a femoral hernia, with a reported incidence of 0.5–5% of femoral hernias; concurrent acute appendicitis is exceptional, with a reported incidence of 0.08–0.13%. Its association with secondary mechanical intestinal obstruction is rarely described in the literature, and preoperative diagnosis remains an ongoing clinical challenge. We report this uncommon presentation in an elderly Peruvian woman.

**Case Presentation:**

An 86-year-old woman presented to the emergency department with a one-day history of colicky abdominal pain accompanied by progressive abdominal distension, nausea, and vomiting of alimentary content. She reported passing flatus per rectum and disclosed a history of drainage of a right inguinal abscess performed one day prior to admission at a private clinic. On examination, she was in a fair general condition with signs of dehydration and moderate subcutaneous tissue. Focused abdominal examination revealed distension with tympanic resonance, metallic bowel sounds, and tenderness over the lower abdomen Laboratory studies revealed a normal leukocyte count (9.95 × 10⁹/L), hyponatremia (Na, 127 mmol/L), hypochloremia (Cl, 88.2 mmol/L), neutrophilia (92%) with a left shift, and a markedly elevated C-reactive protein level (244.7 mg/L). Plain abdominal radiography showed dilated small-bowel loops with multiple air-fluid levels. Contrast-enhanced computed tomography (CT) corroborated signs of intestinal obstruction and additionally identified a femoral mass containing a tubular gas-filled structure within the hernial sac, consistent with De Garengeot hernia complicated by intestinal obstruction.

**Diagnostic Assessment and Outcomes:**

The patient underwent exploratory laparotomy with appendectomy, peritoneal lavage and drainage, adhesiolysis, and primary closure of the femoral hernia defect. The postoperative diagnosis was acute appendicitis complicated by appendiceal abscess in the setting of De Garengeot hernia with secondary intestinal obstruction. Postoperative antibiotic therapy comprised intravenous ceftriaxone and metronidazole. The nasogastric tube was removed on postoperative day three, and the patient was discharged on postoperative day ten without complications.

**Conclusion:**

De Garengeot hernia complicated by appendicitis and intestinal obstruction represents a diagnostic challenge owing to its rarity and the non-specific nature of its clinical presentation. It should be suspected in elderly patients presenting with a painful femoral hernia associated with signs of intestinal obstruction, particularly in obese women, in whom body habitus may impair physical examination and conceal the hernial finding. A high index of clinical suspicion, contrast-enhanced multiplanar abdominopelvic CT, and timely surgical intervention are essential to establish the diagnosis and prevent life-threatening complications.

## 1 Introduction

De Garengeot hernia is defined as the presence of the vermiform appendix within the sac of a femoral hernia. The appendix is found within a femoral hernia in approximately 0.5%–5% of cases, whereas concurrent acute appendicitis within the hernial sac is considerably rarer, with a reported incidence of 0.08%–0.13% (1, 2). Femoral hernias account for less than 5% of all groin hernias and predominate in elderly women, in whom age-related changes to the femoral ring and connective tissue increase the risk of incarceration (1, 3).

Preoperative diagnosis is challenging because the clinical presentation mimics that of an incarcerated or strangulated femoral hernia, and the diagnosis is most frequently established intraoperatively (4). Contrast-enhanced CT has improved preoperative recognition and is considered the imaging modality of choice (5, 6). Reported complications include gangrenous appendicitis and appendiceal perforation, appendiceal abscess, peritonitis, and small bowel obstruction, the latter representing a particularly infrequent complication (7, 8).

We describe the case of an 86-year-old woman with complicated De Garengeot hernia presenting as mechanical intestinal obstruction, one week after drainage of an inguinal abscess. This case is distinctive in combining three uncommon features: appendicitis within a femoral hernia, a preceding inguinal abscess, and secondary intestinal obstruction—in a context where a recent inguinal infection obscured the true underlying diagnosis. The report is structured in accordance with the CARE guidelines (9).

## 2 Case presentation

**Figure 1:**
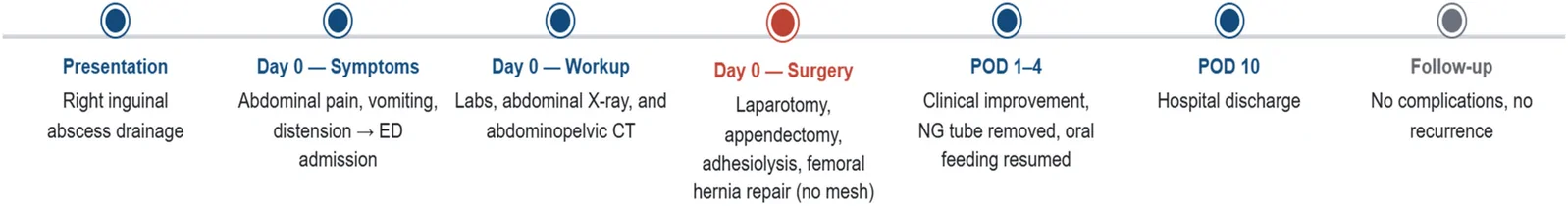
Chronological timeline of the episode of care. The sequence progresses from the antecedent right inguinal abscess drainage, through symptom onset and emergency-department admission (Day 0), diagnostic work-up (laboratory tests, plain abdominal radiography, and abdominopelvic CT), and emergency surgery (laparotomy, appendectomy, adhesiolysis, and femoral hernia repair without mesh), to the uneventful postoperative course (nasogastric tube removal and resumption of oral feeding on postoperative days 1–4), hospital discharge on postoperative day 10, and outpatient follow-up without complications or recurrence.

An 86-year-old woman with no known drug allergies was admitted through the emergency department under the internal medicine service. She had no history of prior abdominal or groin surgery and no relevant family, psychosocial, or hereditary conditions. She reported a pertinent recent history: a right inguinal abscess drained at a private clinic one day prior to admission. She presented with a one-day history of diffuse colicky abdominal pain, associated with progressive abdominal distension and vomiting of alimentary content. She reported passing flatus per rectum and stated that her last bowel movement, normal in character, had occurred one day before presentation. The chronology of the episode of care—from presentation and investigations through surgery, postoperative recovery, and follow-up—is summarized in Figure 1.

On admission, the patient was in a fair general condition with signs of dehydration. Focused abdominal examination revealed a mobile abdomen with moderate subcutaneous tissue; the abdomen was distended and tympanic with metallic resonance on percussion, and there was pain on palpation of the lower abdomen. Bowel sounds were initially hyperactive and subsequently absent. The Guéneau de Mussy sign was positive, compatible with peritoneal irritation and mechanical intestinal obstruction. Cardiovascular assessment documented a blood pressure of 120/70 mmHg and a heart rate of 70 bpm, with a Class II cardiovascular surgical risk classification.

Admission laboratory notable findings included hyponatremia (Na 127 mmol/L), hypochloremia (Cl 88.2 mmol/L). The total leukocyte count was within normal limits (9.95 × 10⁹/L); however, there was neutrophilia (92%) with a left shift (2% band forms) and a markedly elevated C-reactive protein (244.7 mg/L; reference range < 5 mg/L), consistent with an acute inflammatory response. Renal function (creatinine 0.55 mg/dL) and coagulation parameters (INR 0.97) were preserved, and hemoglobin was 10.8 g/dL. An arterial blood gas analysis obtained during surgical observation demonstrated a lactate of 1.5 mmol/L and a bicarbonate of 23.9 mmol/L, with partial electrolyte correction following resuscitation (Na 131 mmol/L, K 4.3 mmol/L). All laboratory values are reported in SI units (10).

**Figure 2:**
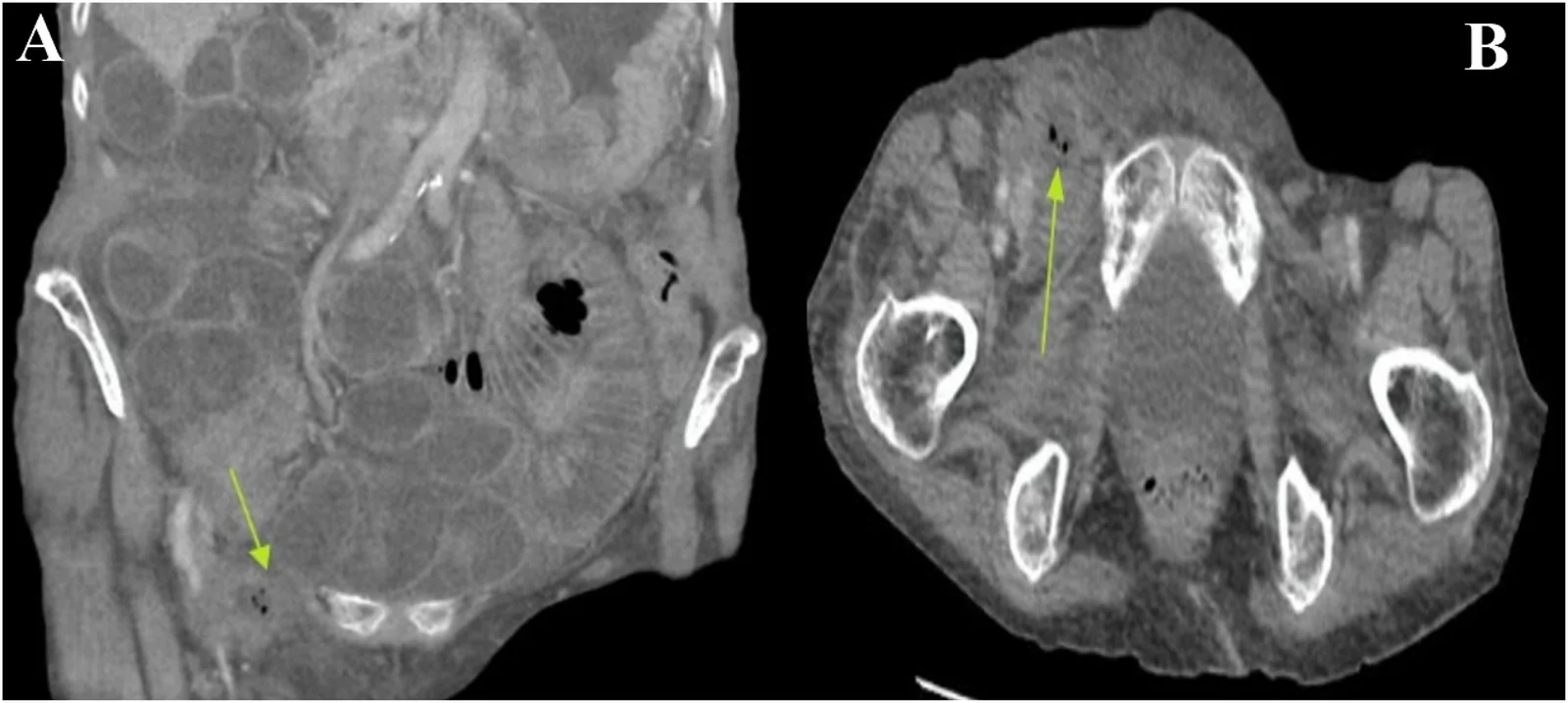
Abdominopelvic CT. **(A)** Coronal reconstruction: dilated small bowel loops with air-fluid levels (intestinal obstruction); the arrow indicates the herniated content within the right femoral defect. **(B)** Axial section at the level of the pubic symphysis: the arrow identifies the herniated content within the right femoral canal, medial to the femoral vessels, with a focus of gas, corresponding to De Garengeot hernia.

Plain abdominal radiography demonstrated dilated small bowel loops with air-fluid levels. Contrast-enhanced abdominal CT confirmed signs of intestinal obstruction and additionally revealed a femoral mass containing a tubular gas-filled structure within the hernial sac, consistent with De Garengeot hernia complicated by intestinal obstruction (Figure 2). The differential diagnosis at the time of surgical evaluation included intestinal obstruction secondary to a femoral hernia. The patient was admitted, and general supportive measures were initiated, focused on stabilization of the internal milieu, with priority given to fluid resuscitation and electrolyte balance management. A nasogastric tube was placed, draining 300 mL of bilious content. The diagnostic challenges were considerable: the previously drained inguinal abscess had obscured the evolving intra-abdominal process, and the definitive diagnosis of De Garengeot hernia was confirmed by imaging and intraoperative findings (11).

## 3 Surgical procedure

**Figure 3:**
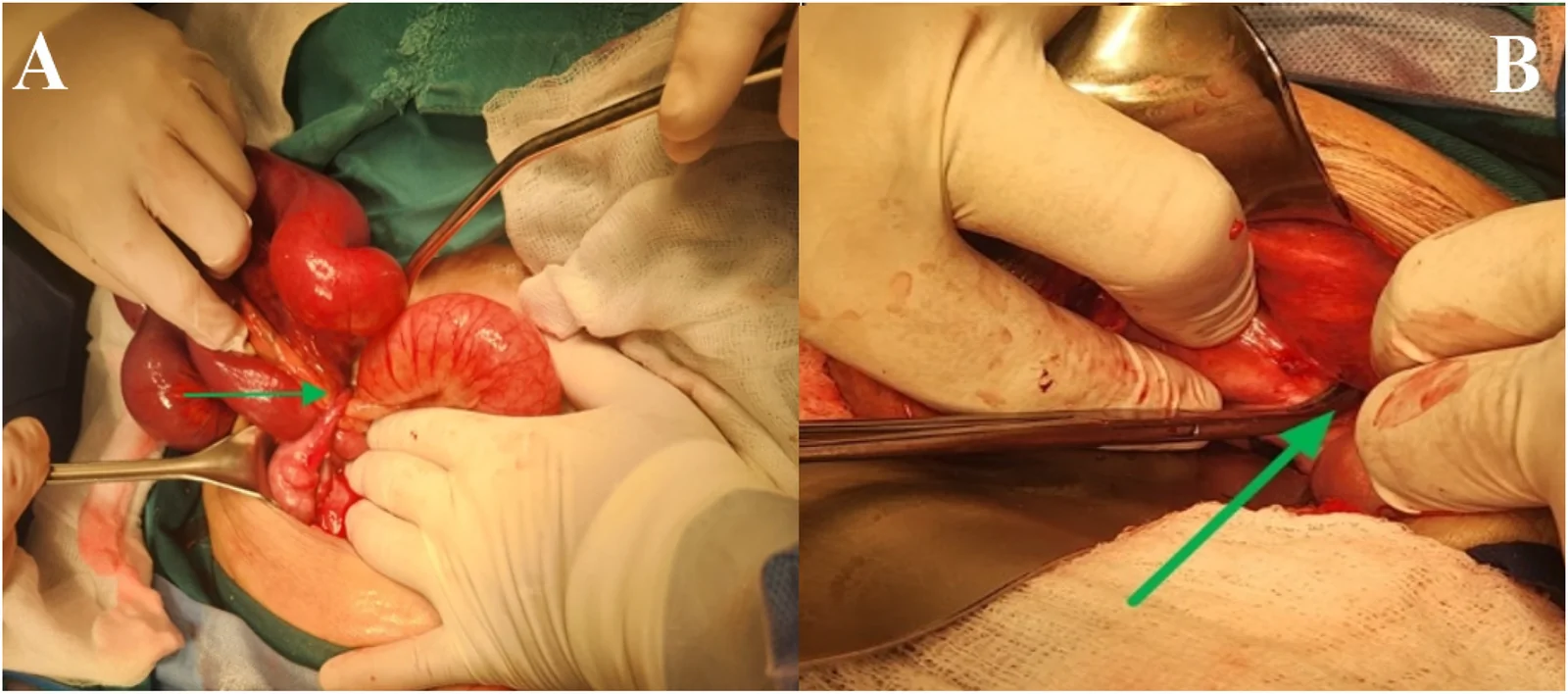
Cecal appendix within the femoral hernia defect (arrow), exhibiting congested serosa and color change consistent with vascular compromise. An adjacent small bowel loop displays macroscopic changes compatible with secondary intestinal obstruction **(A)** Femoral hernia defect after removal of the cecal appendix: the surgical clamp (arrow) traverses the hernial orifice, confirming its location and dimensions prior to repair **(B)**.

Following preoperative risk stratification, fluid and electrolyte resuscitation, nasogastric decompression, and internal milieu management, the patient was scheduled for surgery and underwent exploratory laparotomy. Laparoscopy was considered but was deemed unsuitable in this setting: the marked abdominal distension limited the working space and safe establishment of pneumoperitoneum; the established peritoneal contamination from the appendiceal abscess and the overt mechanical obstruction required direct assessment of bowel viability; and laparoscopic equipment and expertise were not available at the time of this emergency. An open approach therefore offered the most controlled management of the contaminated field and the obstructed bowel. The intraoperative diagnosis was acute appendicitis complicated by appendiceal abscess and De Garengeot hernia with intestinal obstruction (Figure 3A). Intraoperative findings also revealed ileal bowel compromise; however, intestinal viability improved after the application of warm compresses, and bowel resection was therefore not required. The procedure comprised adhesiolysis, appendectomy, peritoneal lavage and drainage, primary closure of the femoral hernia defect with 2–0 nylon (Figure 3B), and placement of a Penrose drain in the pelvic pouch. Mesh was not used because of the contaminated surgical field.

### 3.1 Postoperative course

Postoperative antibiotic therapy consisted of intravenous ceftriaxone 2 g every 24 h, administered from 28 August 2024 to 4 September 2024 (8 days), combined with intravenous metronidazole from 28 August 2024 to 31 August 2024 (4 days). Microbiological cultures of the peritoneal fluid were not available. The immediate postoperative course was favorable. The nasogastric tube was removed on postoperative day three, after which the patient was initiated on a progressive diet that was gradually tolerated, followed by passage of flatus and stool, indicating resumption of gastrointestinal transit. The patient was discharged from the surgical ward on postoperative day ten. Postoperative imaging confirmed the diagnosis of De Garengeot hernia complicated by intestinal obstruction. No adverse events or unanticipated incidents were documented during the hospital stay. During outpatient follow-up, the patient remained asymptomatic, with adequate oral tolerance, restoration of normal bowel function, and no evidence of surgical-site infection or hernia recurrence.

## 4 Discussion

This case illustrates an uncommon convergence of pathologies: acute appendicitis within a femoral hernia (De Garengeot hernia), complicated by appendiceal abscess and secondary mechanical intestinal obstruction in an elderly woman. The epidemiological profile of our patient is consistent with the published literature, in which De Garengeot hernia predominantly affects elderly women, with reported median ages of approximately 78 years and a marked female predominance (1, 12). The reported incidence of acute appendicitis in this context ranges from 0.08% to 0.13%, and concurrent intestinal obstruction is even more exceptional (2). The systematic review by Guenther et al (1) currently represents the largest body of evidence on this entity and underscores that secondary intestinal obstruction is a particularly infrequent and high-risk complication.

### 4.1 Comparison with previously reported cases

To contextualize the rarity of this presentation, we compared our case with 14 previously published reports of De Garengeot hernia (Table 1). In the published literature, the entity occurs predominantly in elderly women and most frequently presents as an isolated, irreducible groin mass; the appendix within the sac is often normal or shows only acute inflammation, and single complications—such as appendiceal perforation, gangrene, or a localized abscess—are described in a minority of cases (13–16). Secondary mechanical intestinal obstruction is conspicuously absent from these reports, and no previously published case has combined acute appendicitis, an appendiceal abscess, and intestinal obstruction within a De Garengeot hernia. This comparison confirms the novelty of the present case, which, to our knowledge, is the first to describe this complete triad, further compounded by a preceding inguinal abscess that masked the underlying diagnosis.

**Table 1:** Comparative summary of previously published De Garengeot hernia cases and the present case.

Study (year)	Age/sex	Clinical presentation	Appendiceal/hernia complication	Surgical management	Outcome
Hayakawa and Hackett, 2015 (17)	83/F	Asymptomatic; incidental finding during laparoscopic cholecystectomy	Incarceration; appendix within femoral sac (incidental)	Laparoscopic identification and hernia management	Uneventful
Misiakos et al. 2018 (18)	Adult/M	Irreducible, tender right groin lump with erythema; no obstruction	Low-grade appendiceal mucinous neoplasm within the sac	Open appendectomy and hernia repair	Favorable
Jin et al. 2016 (19)	58/F	1-week irreducible painful right groin lump with nausea and vomiting	Acute appendicitis (correct preoperative identification)	Appendectomy and femoral hernia repair	Favorable
Caygill et al. 2011 (14)	78/F	24-h painful groin lump	Acutely inflamed appendix with purulent fluid	Open low inguinal incision; appendectomy	Favorable
Coskun et al. 2011 (20)	65/F	3-day painful, non-reducible right groin mass	Inflamed appendix (emphasis on early diagnosis)	Open appendectomy and femoral repair	Favorable
Hao et al. 2014 (21)	81/F	Painful groin swelling	Acute appendicitis (ultrasound/CT diagnosis)	Surgical repair	NR
Gonzalez Alcolea et al. 2017 (22)	85/F	Progressive right groin mass and abdominal pain; no obstruction	Incarcerated femoral hernia with appendix (two cases)	Appendectomy and hernia repair	Favorable
Sinraj et al. 2016 (23)	38/F	Chronic groin swelling with pain and vomiting	Suspected strangulation	Emergency appendectomy and repair	Favorable
Liipo et al. 2015 (24)	60/M	Tender, irreducible right groin mass below old hernioplasty scar	Appendix within femoral sac	Appendectomy and repair	Favorable
Pan et al. 2015 (25)	50/F	4-h sudden painful, irreducible right groin mass	Acute appendicitis requiring early surgery	Appendectomy and hernia repair	Favorable
Bloom et al. 2017 (13)	Elderly/F	1-month generalized abdominal pain, right lower quadrant	Perforated appendix	Operative management; mesh avoided (contaminated)	Favorable
Shiihara et al. 2017 (26)	Adult/F	Irreducible, non-strangulated 3 × 3 cm mass below right inguinal ligament	Acute appendicitis	Combined laparoscopic-open (“two-way”) approach	Favorable
Taveras and Huerta, 2017 (15)	94/M	Acute painful right inguinal bulge	Appendiceal abscess with femoral hernia	Groin exploration under local anesthesia; appendectomy	Favorable
Zainudin et al. 2020 (16)	58/F	24-h nausea, vomiting, painful right groin swelling	Gangrenous appendix	Sac isolation, appendectomy, femoral repair	Favorable

F, female; M, male.

### 4.2 Diagnostic and surgical considerations

Preoperative diagnosis is notoriously difficult, as the presentation overlaps with that of an incarcerated or strangulated femoral hernia, and the majority of cases are identified intraoperatively (1, 5). In our patient, an additional confounding factor was the inguinal abscess drained one day prior to admission, which plausibly represented an early external manifestation of the same underlying process and delayed recognition of the evolving intra-abdominal complication. This masking mechanism has been previously described: Wang et al. (11) reported a case of De Garengeot hernia mimicking an abdominal perforation, and Brain and Barsoom (27) highlighted the importance of maintaining a high index of suspicion in atypical presentations. Contrast-enhanced CT was instrumental in our case in suggesting the diagnosis and distinguishing it from a Richter-type femoral hernia—the principal differential diagnosis at surgical evaluation—consistent with multiple reports positioning CT as the most useful preoperative imaging modality (28, 29).

Surgical management of De Garengeot hernia is not standardized due to its rarity, and the approaches described in the literature vary widely: from open appendectomy with hernia repair through a single inguinal incision (30) and the McEvedy approach (31, 32), to laparoscopy or combined laparoscopic-open surgery (33, 34). More recently, totally laparoscopic techniques such as transabdominal preperitoneal repair (TAPP) with appendix preservation (35, 36) and laparoscopic procedures as the preferred approach at experienced centers have been reported (8, 37). In the context of our patient—with appendiceal abscess, peritoneal contamination, and overt intestinal obstruction—exploratory laparotomy with appendectomy, lavage, drainage, adhesiolysis, and primary closure of the femoral hernia defect without mesh was the appropriate surgical decision, in accordance with the available evidence for contaminated fields (4, 7). Consistent with this evidence, prosthetic mesh is generally avoided in the presence of an appendiceal abscess or perforation because of the elevated risk of infection; primary suture repair or biological materials, which offer better performance in contaminated fields, are preferred.

The ten-day postoperative stay without complications is consistent with the median reported in the systematic review by Guenther et al. (1), in which surgical site infection constitutes the most frequent postoperative complication. The favorable outcome of our patient, despite her advanced age and the severity of the clinical presentation, reinforces that timely surgical intervention—even in high-complexity settings—can achieve satisfactory results in older adults (38, 39).

### 4.3 Strengths and limitations

The main limitation is inherent to the nature of a single case report, which precludes generalization of findings or estimation of incidence, risk factors, or comparative outcomes. Moreover, as the diagnosis was established in an urgent surgical context, some preoperative diagnostic elements were necessarily limited. Nevertheless, the case provides an important clinical lesson: De Garengeot hernia should be considered in the differential diagnosis of elderly patients presenting with a groin mass, abdominal pain, or features of intestinal obstruction, particularly when recent inguinal infection or local inflammation may mask the underlying pathology. Early contrast-enhanced CT, prompt surgical exploration, and readiness to adapt the operative strategy remain essential to achieving a favorable outcome.

## 5 Conclusion

De Garengeot hernia simultaneously complicated by acute appendicitis, appendiceal abscess, and secondary intestinal obstruction is an extremely rare surgical entity for which a definitive preoperative diagnosis is not always attainable. In the present case, contrast-enhanced abdominopelvic CT demonstrated intestinal obstruction associated with a right femoral hernia, but surgical exploration established the definitive diagnosis and revealed the triple concurrence. A preceding inguinal abscess drainage acted as a clinical distractor that delayed recognition of the true emergency; a prior inguinal infection does not exclude, and may in fact mask, a complicated femoral hernia containing the appendix. We therefore recommend considering De Garengeot hernia in elderly patients presenting with intestinal obstruction, a right femoral hernia, and a concomitant or recent inguinal abscess, even when CT does not identify the appendix within the sac. In such cases, early surgical exploration is both the definitive diagnostic tool and the most effective treatment.

### 5.1 Patient perspective

Owing to her acute clinical condition and disorientation at admission, the patient was unable to provide a detailed personal account during the initial episode. At discharge, she reported complete resolution of the abdominal pain, nausea, vomiting, and distension. She described the initial period as frightening, particularly because the groin swelling and abdominal symptoms had progressed rapidly over a single day, but she felt reassured by the explanations provided by the surgical team and by the speed with which she was taken to the operating room. She expressed satisfaction with the treatment and gratitude for the care provided by the multidisciplinary team. During outpatient follow-up, she reported a progressive recovery, with good tolerance of oral intake, restoration of normal bowel habits, and a gradual return to her usual daily activities without recurrence of symptoms. She stated that the timely surgical intervention had allowed her to regain her well-being and improve her quality of life, and that she would not have expected such a rapid recovery given her age.

## Data Availability

The raw data supporting the conclusions of this article will be made available by the authors, without undue reservation.
